# Supplementary material for: HDAC Inhibition Increases HLA Class I Expression in Uveal Melanoma
Source: Cancers (Basel). 2020 Dec 9;12(12):3690. doi: 10.3390/cancers12123690 (PMC7763827; doi:10.3390/cancers12123690)
Supplement: Supplementary file 1 [file cancers-12-03690-s001.pdf]

cancers

MDPI

Supplementary materials

HDAC Inhibition Increases HLA Class I Expression in Uveal Melanoma

Zahra Souri [1], Aart G. Jochemsen [2], Mieke Versluis [1], Annemijn P.A. Wierenga [1], Fariba Nemati [3], Pieter A. van der Velden [1], Wilma G.M. Kroes [4], Robert M. Verdijk [5,6], Gregorius P.M. Luyten [1] and Martine J. Jager [1,*]

[1] Department of Ophthalmology, LUMC, Albinusdreef 2, 2333 ZA Leiden, The Netherlands; z.souri@lumc.nl (Z.S.); m.versluis@lumc.nl (M.V.); a.p.a.wierenga@lumc.nl (A.P.A.W.); p.a.van_der_velden@lumc.nl (P.A.v.d.V.); g.p.m.luyten@lumc.nl (G.P.M.L.)
[2] Department of Cell and Chemical Biology, LUMC, 2333 ZA Leiden, The Netherlands; a.g.jochemsen@lumc.nl
[3] Laboratory of preclinical investigation, Department of Translational Research, Institut Curie, PSL University, 75248 Paris, France; fariba.nemati@curie.fr
[4] Department of Clinical Genetics, LUMC, 2333 ZA Leiden, The Netherlands; w.g.m.kroes@lumc.nl
[5] Department of Pathology, LUMC, 2333 ZA Leiden, The Netherlands; r.m.verdijk@lumc.nl
[6] Department of Pathology, Section Ophthalmic Pathology, ErasmusMC, Dr Molewaterplein 40, 3015 GD Rotterdam, The Netherlands
* Correspondence: m.j.jager@lumc.nl

Received: 12 November 2020; Accepted: 07 December 2020; Published: 09 December 2020

Supplementary materials

Table S1. Gene expression (Illumina) Probe Number in an Illumina HT12v4 array and mean expression of the genes used in this study.

Gene of interest	Illumina probe number	Mean expression ± SD
**HLA-A probe 1**	ILMN_1671054	11.40 ± 0.90
**HLA-A probe 2**	ILMN_2203950	13.84 ± 0.77
**HLA-A probe 3**	ILMN_2186806	10.71 ± 1.45
**HLA-B**	ILMN_1778401	11.33 ± 1.67
**HDAC1**	ILMN_1727458	10.18 ± 0.45
**HDAC2**	ILMN_1767747	9.28 ± 0.48
**HDAC3**	ILMN_1772455	8.49 ± 0.20
**HDAC4**	ILMN_1764396	7.57 ± 0.39
**HDAC5 probe 1**	ILMN_2388166	6.52 ± 0.10
**HDAC5 probe 2**	ILMN_1810856	6.63 ± 0.13
**HDAC6**	ILMN_1798546	7.94 ± 0.26
**HDAC7 probe 1**	ILMN_3266186	6.86 ± 0.20
**HDAC7 probe 2**	ILMN_1728521	6.89 ± 0.21
**HDAC8**	ILMN_1651544	6.85 ± 0.24
**HDAC9**	ILMN_2408885	6.68 ± 0.12
**HDAC11**	ILMN_1684690	7.49 ± 0.40
**EZH2**	ILMN_1708105	6.65 ± 0.15

Table S2. Sequences of PCR primers used in this study.

Primers	Forward	Reverse
**HLA-A**	5'- TGTGTTCGTGTAGGCATA	5'- TTGAGACAGAGATGGAGAC
**HLA-B**	5'- CTCCATCTCTGTCTCAACTT	5'- CATCAACCTCTCATAGCA
**RPS11**	5'- AAGCAGCCGACCATCTTTCA	5'- CGGGAGCTTCTCCTTGCC
**CAPNS1**	5'- ATGGTTTTGGCATTGACACATG	5'- GCTTGCCTGTGGTGTCGC
**SRPR**	5'- CATTGCTTTTGCACGTAACCAA	5'- ATTGTCTTGCATGCGGCC
